# The social cost of carbon driven by green behaviors

**DOI:** 10.1371/journal.pone.0286534

**Published:** 2023-06-30

**Authors:** Min Fu, Yixiang Zhang, Lixin Tian, Zaili Zhen

**Affiliations:** 1 Research Institute of Carbon Neutralization Development, School of Mathematical Sciences, Jiangsu University, Zhenjiang, Jiangsu, P.R. China; 2 Jiangsu Province Engineering Research Center of Spatial Big Data, School of Mathematical Sciences, Nanjing Normal University, Nanjing, Jiangsu, P.R. China; 3 Jiangsu Province Engineering Research Center of Industrial Carbon System Analysis, School of Mathematical Sciences, Jiangsu University, Zhenjiang, Jiangsu, P.R. China; 4 Key Laboratory for NSLSCS, Ministry of Education, School of Mathematical Sciences, Nanjing Normal University, Nanjing, Jiangsu, P.R. China; Shenzhen University, CHINA

## Abstract

With the change of climate issues and the needs of economic development, the idea of practicing green and low-carbon behaviors sinks deeper and deeper into people’s hearts. This paper based on the social cost of carbon (SCC) model, this paper constructs a new carbon social cost model by adding the impact of green low-carbon behavior. Classify climate states, based on Bayesian statistical knowledge, study the posterior probability distribution of climate state transitions, and discuss the optimal carbon policy for different climate states by balancing emission utility costs and utility weighted carbon marginal products. This article also discusses the damage caused by rising temperatures and explores their impact on carbon price policies. then, the paper calculates SCC under four kinds of climate states, which will be visually displayed with graphs. Finally, we compare SCC obtained in this paper with that in other researches. The results show that: (1) Climate status has a significant impact on carbon policy, and carbon price predictions will dynamically change with climate status. (2) Green low-carbon behavior has a positive impact on climate status. (3) There are differences in the impact of the three types of damage caused by rising temperatures on carbon price policies. (4) Green development is conducive to stabilizing the value of SCC. (5) Close monitoring of the climate state helps to update the probability of damage in time so that we can precisely adjust the corresponding policies on SCC. This study provides theoretical and empirical reference for the government to formulate carbon price policies and promote the development of social green behavior.

## 1. Introduction

Climate change is a global issue, and extreme climate events caused by global warming have become a new threat to international peace. The global destiny is shared and no country in the world can stand alone. Only the international community work together, the issue of climate change can better deal with [1]. One of the main sources of climate change is excessive emission of greenhouse gases, which is directly related to carbon emission behaviors. Carbon emissions have externalities, which is difficult to quantify climate changes. Pigou proposed the use of taxes to solve the problem of externalities and the concept of carbon taxes to internalize the external costs of carbon emissions [2]. As people are concerned about the environment, the perception of climate states is constantly updated, a research report issued by the United Nations Intergovernmental Panel on Climate Change (IPCC) pointed out that a temperature rise within 1.5 degrees Celsius will not endanger the habitability of the earth [3].

To assess SCC, we mainly apply to Integrated Assessment Models for Climate, which can be classified into three categories according to the method used: optimization models, computable general equilibrium models (CGE models), and simulation models [4]. A representative model in optimization modeling is the Dynamic Integrated Climate Economy (DICE). Nordhaus W D [5] used the DICE model to calculate SCC by analyzing the economic and environmental impacts of alternative policies using a simple and easy-to-understand approach, and concluded that the losses caused by climate change increase as output decreases. The carbon cycle model in the DICE model is an important prerequisite for estimating SCC and has undergone refinement from an early single-tier carbon pool [6] to a three-tier carbon pool [7]. Bijgaart et al. [8] studied a box carbon cycle system in a continuous state of time and approximated SCC using a marginal cost approach. Michael D G. et al. [9] used a dynamic stochastic general equilibrium model adapted from the DICE model and found that a relaxed “policy slope” mitigation strategy is preferable to a more aggressive mitigation strategy. Botzen W J W. et al. [10] used an alternative approach to calculate the optimal climate policy for the DICE model and showed that the optimal mitigation policy is very sensitive to climate damage. Dayaratna. et al. [11] balanced the climate sensitivity distribution by new estimates in the DICE model and found that appropriate sensitivity parameter values can effectively reduce the uncertainty. The simulation model is primarily used to assess SCC under a variety of possible future emission scenarios. Hope C. [12] applied the PAGE (Policy Analysis of the Greenhouse Effect) model to give the costs of multiple scenarios and optimal pathways. Stanton E A. et al. [13] evaluated SCC in a multi-emissions scenario. Pizer W A. et al. [14] incorporated both potential long-term losses from climate change and the cost of greenhouse gas abatement into a computable general equilibrium model and showed that the price mechanism scheme outperformed the quantity mechanism scheme and that the efficiency of the hybrid mechanism scheme would be improved. Adao B. et al. [15] studied the impact of technological progress on the optimal transition to a renewable energy driven world economy.

In addition, studies exploring the future SCC, which combine elements of technological progress, green behaviors, and probability, have received increasing attention in recent years. Dietz [16] considered damages from sea level rise in the probabilistic comprehensive assessment model to empirically test the key theories in the study and expressed the key parameters of economic costs and climate sensitivity with the help of the thick-tailed distribution. Golosov et al. [17] innovated the neo-classical model and divided the carbon tax into a fixed tax and a time-varying tax for oil, indicating that the optimal strategy is robust to uncertainty. Gerlagh R., Liski M. [18] provide a detailed description of learning dynamics and the emission-temperature response under the description of the global carbon cycle in a climate economic model. Pycroft J. et al. [19] estimated SCC and assessed the probability of losses from climate change by using a comprehensive evaluation model based on economic and climate systems. This paper helps to show the uncertain climate impacts and related estimates of SCC in the integrated system model. Cai Y. et al. [20] established a nine-dimensional dynamic optimization question which was presented by Bellman equation to discuss SCC under a variety of abrupt climate change scenarios, concluding that SCC would fluctuate greatly due to economic or climate risks. Bourgeon J M. and Hovsepian [21] analyzed the use of green technology in a dynamic economy affected by stochastic impact. Gerlagh R., Liski M. [22] studied the optimal future pricing of SCC when the effects of climate change are uncertain, and a quantitative assessment suggests that the price of carbon will grow roughly at the economic growth rate over the next 100 years. Wan B. et al. [23] expressed the diffusion of green low-carbon behaviors in terms of the amount of knowledge diffusion and proposed and compared three regimes of green low-carbon behaviors. Besides, some scholars used the meta-analysis method to explore the factors affecting the green behavior of enterprises, government and rural areas, exploring their behavioral mechanisms [24–26]. Sujahangir K S et al. [27] analyzed the future SCC under two emission scenarios and derived the optimal policy by using Malaysia as a starting point to achieve the emission reduction target by 2050. Zhen Z, Tian L. [28] compared the effects of different climate damage functions and different values of temperature increase on SCC. Zeng Hui fang and Xiong [29] explored the effect of prior information on SCC in a Bayesian statistical approach, and the correction can make SCC less affected by the prior information. Stern N, Stiglitz J E. [30] argued that the flaws in the IAMs model would overestimate SCC. While Khanna M et al. [31] studied the impact of repealing the Clean Power Plan on SCC. Larry S. K et al. [32] constructed a new type of cap-and-trade system to pool information through market prices in order to eliminate the uncertainty caused by asymmetric information.

It can be seen that the researches above mainly study SCC through comprehensive climate assessment models, including temperature rise risk, different carbon emission scenarios, discount rates, and extreme disasters. There are few studies on the structure of SCC with green behaviors, and not many of them combine the knowledge related to Bayesian statistics to study the climate states. In addition, a major reason for the elevated SCC estimates is the elevated damage function [20, 33], but there is few researches that analyze the damage of temperature rise utility. The differences between the paper and existing studies are shown in Table 1.

**Table 1:** Research progress on the social cost of carbon.

Literature	Green low -carbon behaviors	Temperature rise damage	Climate models	Classification of climate states	Carbon cycle	Combined with Bayesian statistics
Dietz [16]	No	Yes	No	No	Linear	No
Golosov et al. [17]	No	Yes	No	No	Linear	No
Gerlagh R. et al. [18]	No	Yes	No	No	Linear	No
Pycroft J. et al. [19]	No	No	No	No	No	Yes
Cai Y et al. [20]	No	Continuous	Yes	2 kinds	Linear	No
Bijgaart et al. [8]	No	Yes	No	No	Linear	No
Gerlagh R. et al. [18]	No	Discrete	Yes	3 kinds	Linear	Yes
Zhen Z. et al. [28]	No	Discrete	No	2 kinds	No	No
This paper	Yes	Discrete	Yes	4 kinds	Nonlinear	Yes

Therefore, considering the realistic research context and theoretical research development, this paper presents the core research question: From the perspective of people’s green behavior, how to calculate SCC? This paper explores and construct a SCC model driven by green behaviors from three modules. On the level of economic module, the value of green behaviors is represented by the amount of green behaviors output, and the accounting of gross product takes into account consumption, investment, and production losses due to temperature rise. The utility module takes into account consumption, environmental quality, and the loss of utility due to temperature rise. On the level of climate module, a stochastic probability model of climate based on the carbon cycle and climate damage function is established to classify climate states into four types and to study the corresponding SCC under different climate states.

**Fig 1:**
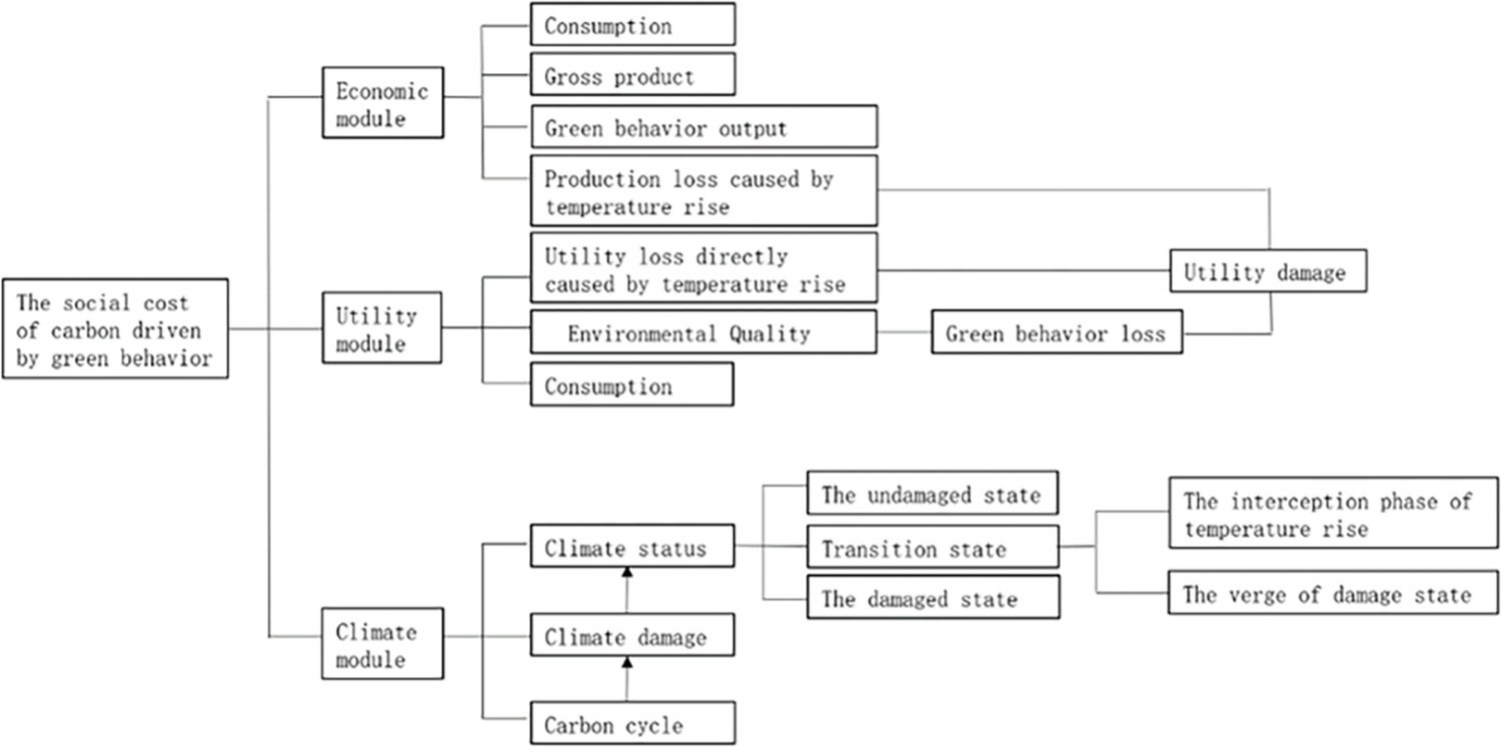
The research framework of this paper.

The research framework of this paper is shown in Fig 1:

This study constructs a new carbon social cost model from three modules of economy, utility, and climate to study the best carbon social cost under different climate conditions. The output of green behavior is constructed in the economic module to describe the value generated by green behavior, and the setting of other functions and the interaction between functions are considered more comprehensively. In the utility module, in addition to considering the two indicators of consumption and the loss of utility caused by temperature rise, this article also considers the impact of environmental quality on people’s happiness. In the climate module, the detected temperature rise is used to measure whether the temperature rise has an impact on economic development.

Compared with previous studies, the innovation of this paper is mainly shown in the following four aspects:

1. From research perspective: this paper constructs the output of green behaviors based on the climate change information dissemination model as a way to characterize the positive effects of green behaviors on economic development and environmental quality and to consider the multifaceted effects of green behaviors on economy, utility and climate.
2. From research design: in the assessment of SCC, this paper also considers the marginal utility cost in addition to the economic marginal cost. The marginal loss of carbon emissions to economy and utility is considered in SCC. Besides, this paper increases the utility damage of current carbon emissions to three aspects, and discusses the impact of these three aspects of utility damage on the estimated damage to SCC. Based on previous studies, we reasonably assume different forms of distribution to discuss in the form of mathematical expectations in the undamaged climate state.
3. From analysis methods: based on the carbon cycle system with nonlinear structure and climate damage function, this paper considers the uncertainty of climate states and obtains the updated value of probability of temperature rise damage through Bayesian statistical method. This method makes up for the deficiency that SCC is difficult to change with the climate states, and provides a more accurate and flexible analysis method for the estimation of SCC.
4. From research conclusions: We found that SCCS change smoothly with four climate states, SCC increases most moderately in the undamaged state, followed by the interception phase of temperature rise. The increase of SCC in the verge of damage state is slightly larger, but it is much smaller than that in the damaged state.

This paper provides more theoretical explanations for the carbon price in the environment of green behavior. This study provides theoretical and empirical reference for the government to better set carbon price. In addition, this article uses a new perspective of thinking to promote the green development of society.

## 2. Model construction

Based on the perspective of people practicing green behaviors, this paper establishes a novel model of SCC through the study of economic module, utility module and climate module. Compared with previous studies, this paper has three aspects of improvement: constructing the output of green behaviors to represent the economic value of green behaviors, and nesting it into the utility module and climate module; obtaining the updated value of the probability of temperature rise damage by using Bayesian methods; and considering SCC based on a combination of the marginal economic cost and the marginal utility cost.

Based on the Cobb-Douglas production function and incorporating the production loss due to temperature rise into the production structure [22], the equation for the gross product is obtained as

$$y_{t}=k_{t}^{\alpha }{\left[A_{t}(l_{y,t},e_{t})\right]}^{\beta }w(s_{t}),$$ (1)

where *k*_*t*_ represents capital, *A*_*t*_(*l*_*y*,*t*_,*e*_*t*_) represents the labor-energy compound function and *α*, *β* are coefficients of elasticity. For the convenience of general analysis, we set *α*+*β* = 1.

The gross production value can be written as:

$$y_{t}=c_{t}+k_{t+1}.$$ (2)

Based on the research of Gerlagh R and Liski [18], assuming a constant share of investment in GDP of *g*, 0<*g*<1, we have

$$k_{t+1}=gy_{t}.$$ (3)

Combined with the construction of Ou X. et al. [34] for climate change information dissemination model, this paper quantifies the impact of green behaviors on improving environmental quality and delaying temperature rise, and constructs the green behaviors stop loss quantity *G*_*t*_, whose expression is

$$G_{t}=G_{0}L\sum_{\tau =0}^{t}\frac{1}{1+(\frac{1}{i_{0}}-1)e^{‐\kappa \tau }}\frac{\tau }{\tau +x}\Delta_{G}{}^{D_{t}},$$ (4)

where *Y* is the output per person, *L* is population size, *i*_0_ is the proportion of people practicing green behaviors in the early stage, *κ* is the spread rate of green behaviors, which is the endogenous growth parameter of green behaviors extraction, and there is no regression of green behaviors diffusion because the green behaviors diffusion parameter set here is exponential. Coefficient $\frac{1}{1+(\frac{1}{i_{0}}-1)e^{‐\kappa \tau }}$ represents the proportion of people practicing green behaviors at *τ* (time), and *x* represents the difficulty of communication in different countries or regions, that is, the difference in the speed of transformation. The smaller the value, the higher the economic level of the country or region, the less difficult the diffusion of green behavior and the faster the diffusion rate. $\frac{\tau }{\tau +x}$ indicates the lag of people’s cognition of green low-carbon concept to the practice of green behaviors in the process of its transmission. Δ_G_≥0 characterizes the loss rate of green behaviors, which represents the extent to which people engage in green and low-carbon behaviors that affect climate change and thus lead to a reduction in climate damage.

This paper incorporates the positive impact of green behaviors on climate change, that is, green behaviors can improve the climate damage, with a climate damage function of

$$D_{t}=\sum_{\tau =1}^{\infty }R_{D,\tau }E_{t‐\tau }-\upsilon G_{t},$$ (5)

where *R*_*D*,*τ*_ is “emission temperature response” and *E*_*t-τ*_ is the historical carbon dioxide emissions. Climate damage is linearly related to historical carbon dioxide emissions, but the green behaviors improve it to some extent. The improvement elasticity factor is denoted by *υ*, and takes different values according to different climate states. *υG*_*t*_ indicates the stop loss of climate damage from green behaviors.

Combined with the research of Gerlagh R, Liski M [22], it is assumed that the loss of production caused by temperature rise depends on the previous emission of fossil fuel.

$$w(s_{t})=e^{-\Delta_{y}D_{t}},$$ (6)

where Δ_y_≥0 is output loss and *D*_*t*_ is climate damage. Thus, the marginal loss caused by carbon dioxide emission at *t* (time) for *t*+*τ* (time) can be obtained as

$$R_{D,}{}_{\tau }=\frac{dD_{t+\tau }}{dE_{t}}=\pi \varepsilon \sum_{i\in L}a_{i}\frac{{\left(1-\eta_{i}\right)}^{\tau }-{\left(1-\varepsilon \right)}^{\tau }}{\varepsilon -\eta_{i}}(1-\frac{2E_{t}}{nk_{e}}).$$ (7)

The emission-temperature response *R*_*D*,*τ*_ represents the dependence of future climate damage *D*_*t+τ*_ on current emission *E*_*t*_, and (1−*η*_*i*_)^*τ*^, (1−*ε*)^*τ*^ represent the delay of carbon concentration and temperature adjustment respectively.

The carbon cycle models are constantly improved, and a three-layer carbon pool model is usually used now. Tian L et al. [35] improved the structure of carbon cycle as a non-linear relationship with

$$\begin{array}{l} S_{i,t+\tau }=a_{i}E_{t+\tau -1}(1-\frac{E_{t+\tau -1}}{nk_{e}})+(1-\eta_{i})a_{i}E_{t+\tau -2}(1-\frac{E_{t+\tau -2}}{nk_{e}})+(1-\eta_{i}{)}^{2}a_{i}E_{t+\tau -3}(1-\frac{E_{t+\tau -3}}{nk_{e}})\\ \;+\cdots +{\left(1-\eta_{i}\right)}^{\tau -1}a_{i}E_{t}(1-\frac{E_{t}}{nk_{e}})+\cdots +{\left(1-\eta_{i}\right)}^{t+\tau -1}S_{i,1}, \end{array}$$ (8)

where *i* is the *i*^*th*^ climate box and *i*∈*I* = {1,2,⋯,*n*}, *S*_*t*_ is the total atmospheric carbon dioxide storage exceeding the industrial storage level at *t* (time), *s*_*i*,*t*_ is the atmospheric carbon dioxide storage in the *i*^*th*^ climate box, *E*_*t*_ is the amount of carbon dioxide emissions at *t* (time), *a*_*i*_ represents the share of carbon emissions that enters each climate box, *η*_*i*_ is the carbon dioxide decay rate of the box *i*. And $1‐\frac{E_{t}}{nk_{e}}$ represents the potential share of carbon dioxide emissions. The conversion process of carbon dioxide emissions from fossil fuels to the concentration of carbon dioxide in the atmosphere is nonlinear. *k*_*e*_ is related to the time period and represents the maximum estimator of carbon emissions in a certain time period. *k*_*e*_ is taken as the estimated value of total carbon emissions before 2100. *n* is the evolution coefficient of carbon dioxide emissions, and its value is related to emission reduction technology, policy impact and other factors. The share of emission growth potential is affected by the change of some factors such as science and technology progress, emission reduction technology, as well as the policy regulation and systematic external factors which affect carbon emissions.

Therefore, the carbon dioxide increments at *t*+*τ* (time) caused by a unit of carbon dioxide emitted at *t* (time) is shown as follows:

$$\frac{dS_{t+\tau }}{dE_{t}}=\frac{d\sum_{{}_{i\in I}}S_{{}_{i,t+\tau }}}{dE_{t}}={\left(1‐\eta_{i}\right)}^{\tau -1}a_{i}(1‐\frac{2E_{t}}{nk_{e}}).$$ (9)

In the setting of utility function, other scholars often used consumption to measure studies based on the law of diminishing marginal utility [36, 37]. In addition, they considered the direct damage of carbon emissions to utility in the evaluation of SCC [22, 38]. However, with the prominent problem of climate change, the quality of environment can increasingly influence people’s well-being. Therefore, this paper uses green behaviors output to evaluate environmental quality and includes the environmental quality in the utility function. So, on the utility level, we consider consumption, environmental quality and direct utility damage caused by temperature rise. So, we have:

$$u_{t}=\omega_{1}\ln c_{t}+\omega_{2}\ln G_{t}-\Delta_{u,t}D_{t},\Delta_{u,t}=\Delta_{u}M_{t},$$ (10)

where *ω*_1_ and *ω*_2_ are weights and *ω*_1_+*ω*_2_ = 1. *M*_*t*_ is defined as the climate state variable. When the climate state varies, the loss of utility caused by temperature rise is also different. Δ_*u*_≥0 characterizes the utility damage.

Previous studies show that global average temperature affects economic development [39]. Based on the previous classification of climate states [22, 33], this paper will classify climate modules in a more detailed way.

We set climate state quantity as *M*_*t*_ and climate state transition probability as *p* which has prior probability *μ*_0_ and posterior probability *μ*_*t*_. The influence of temperature on economic development is reflected by climate state *M*_*t*_, in which the two values 0 and 1 of *M*_*t*_ represent the undamaged state and the damaged state respectively, and the initial value is 0. There are two kinds of transition states: the stage of temperature rises interception and the stage of damage approaching. *M*_*t*_ can be transferred in different states and be expressed by transition probability *p*. We set the prior distribution of probability *p* as two-point distribution, *p* = 0 or *p* = *λ*, and mark *λ* as risk transfer rate.

**Fig 2:**
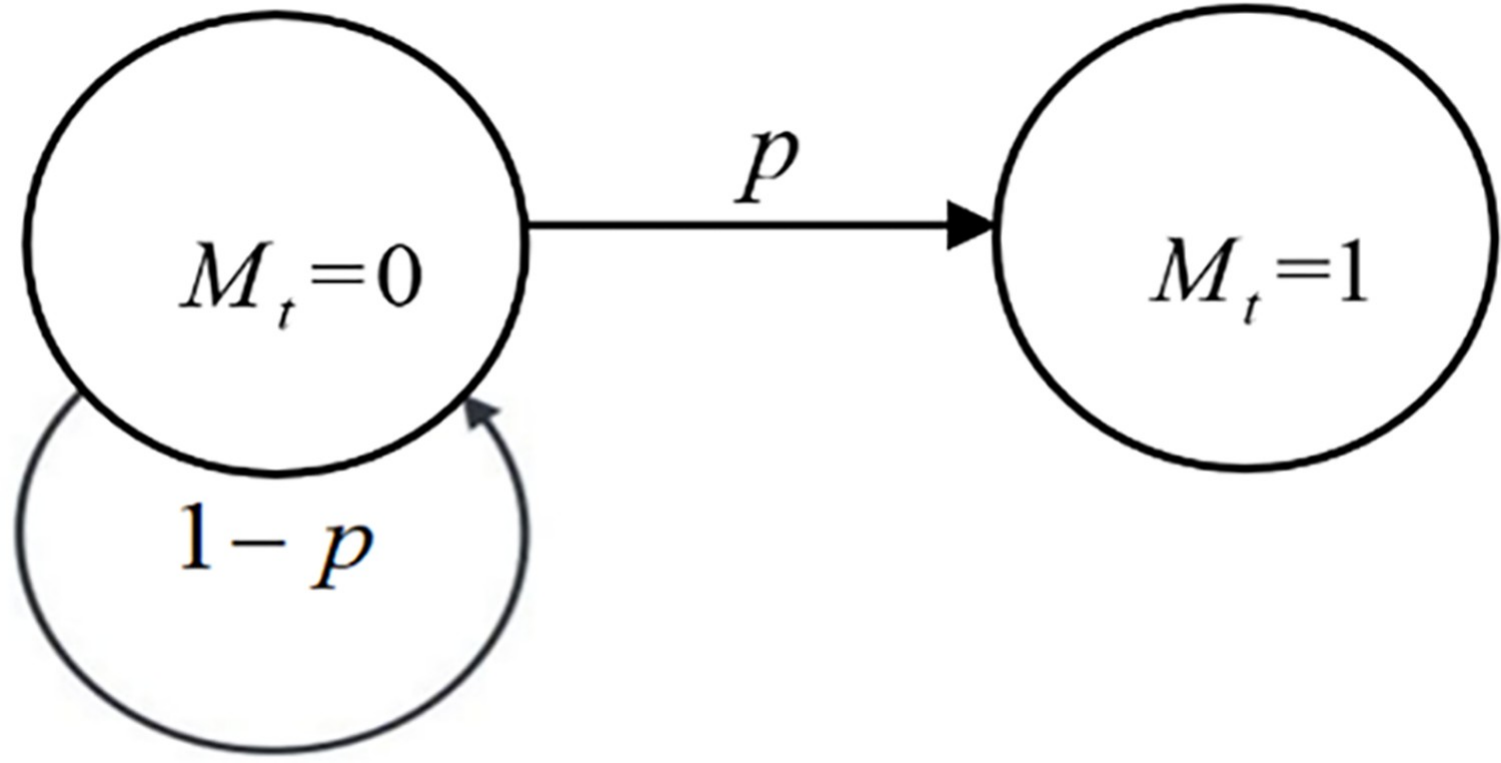
Transfer between different climate states.

The transition between different climatic states can be represented as Fig 2.

Transition probability *p* represents the transition probability in different climate states, and the relationship between climate states and transition probability can be expressed as Table 2.

**Table 2:** Probability of transition between different climate states.

Transition probability of *M*_*t*_	*M*_*t*_ from 0 to 1	*M*_*t*_ remains at 0
*p*	0	1
	*λ*	1−*λ*

The distribution of climate state transition probability can be shown in Table 3.

**Table 3:** Distribution of probability of climate state transition.

*p*	Prior probability	Posterior probability
*p* = 0	1−*μ*_0_	1−*μ*_*t*_
*p* = *λ*	*μ*_0_	*μ*_*t*_

This paper assumes that the probability of damage occurrence *μ* follows the distribution of *β*. The updating rule of posteriori probability *μ*_*t*_ of temperature rise damage is that the initial prior value *μ*_0_~*β*(*α*,*β*) is given, *α* is the number of vigilance, *β* is the number of stable development, and the initial values of *α*, *β* are given according to the initial probability value *μ*_0_. When the climate state changes, the values of parameter *α*, *β* will change accordingly. The posterior probability *μ*_*t*_ of temperature rise damage in each period will be updated.

In the undamaged stage (the temperature rise does not affect the economy in this period, and the assessment of the damage caused by the temperature rise becomes more optimistic), the number of vigilance *α* remains unchanged, the number of stable development *β* will increase gradually, *μ*_*t*_ will decrease with the passage of time, and *μ*_*t*+1_<*μ*_*t*_.

When the accumulated value of temperature rise reaches the set sensitive value, it will enter the transition state. The transition state can be divided into: (1) the interception stage of temperature rise, which means that people reverse the trend of temperature change by practicing green behaviors and energy substitution to remove the possible damage caused by temperature rise to economic development. Due to people’s cautious, the number of vigilances *α* is set to be updated with 90% of the updated information, and the number of smooth developments *β* is updated with 10% of the updated information. In this stage, the transition probability of climate state *p* is taken as *λ*. As time goes on, *μ*_*t*_ will first rise and then fall until the accumulated value of temperature rise falls below the set sensitive value of temperature rise; (2) the damage imminent stage, which means that the damage of climate to economy occurs with a certain probability. In this stage, the number of vigilance *α* increases gradually, while the number of stable development *β* remains unchanged, so the update value of damage probability *μ*_*t*_ will increase with the passage of time, that is, *μ*_*t*_>*μ*_*t*-1_ until the damage occurs. When the damage occurs in period *t*, the climate state is *M*_*t*_ = 1 and is also updated to the damage stage, and the probability of damage occurrence is *μ*_*t*_ = 1.

According to the setting of the probability of climate state transfer, the following conditions can be deduced:

(1) The probability of climate state *M*_*t*_ = 0 is shown as follows:

$$\begin{array}{l} Pr(M_{t}=0)=\mathrm{Pr}(M_{t}=0|p=\lambda )+\mathrm{Pr}(M_{t}=0|p=0)\\ \;=\mu_{0}{\left(1‐\lambda \right)}^{t}+(1‐\mu_{0}){\left(1‐0\right)}^{t}\\ \;=\mu_{0}{\left(1‐\lambda \right)}^{t}+(1‐\mu_{0}), \end{array}$$ (11)

*p* = *λ* or *p* = 0 at *M*_*t*_ = 0, so the above formula indicates that the climate state has not caused damage to the economy at *t* (time).

(2) The probability of period *t* in the interception state of temperature rise is:

$$\mathrm{Pr}(p=\lambda \cap M_{t}=0)=\mu_{0}{\left(1‐\lambda \right)}^{t},$$ (12)

It means that the climate state did not cause damage to the economy in period *t* (time), but the temperature detection value *T* exceeded the set temperature alert value $\overset{•}{T}$, so people controlled or reversed the trend of temperature rise through a series of measures such as increasing green behaviors.

(3) The damage probability of period *t* is:

$$Pr(p=\lambda |M_{t}=0)=\frac{\mu_{0}{\left(1‐\lambda \right)}^{t}}{\mu_{0}{\left(1‐\lambda \right)}^{t}+1‐\mu_{0}},$$ (13)

Eq (13) indicates there is a probability of damage, although the climate state does not cause damage to the economy in the period of *t*.

## 3. Social cost of carbon

Marginal economic cost was usually considered in the previous studies on SCC. This paper also considers marginal loss of utility, that is, to find the optimal SCC by balancing the marginal cost of carbon between economy and utility. The expressions of optimal SCC at *M*_*t*_ = 1 and *M*_*t*_ = 0 are given below, and the estimated damage changes under two climate transition states are explained.

### 3.1. The social cost of carbon in the state of damage

When the damage state *M*_*t*_ = 1, we first work out *L*, the present value of utility marginal cost of current carbon emission, and the economic marginal cost and utility marginal cost of current carbon emission are balanced. We find the corresponding SCC through $\frac{\partial y}{\partial E}\frac{\partial \mu }{\partial C}=L$. For convenience, set Δ as the total degree of utility marginal cost (utility loss), that is, set Δ as the discount value of the derivative of utility *μ*_*t*_ to climate damage *D*_*t*_. Therefore, the utility loss of carbon emission in period *t* is:

$$\Delta =-\sum_{\tau =0}^{\infty }\delta^{\tau }\frac{d\mu_{t+\tau }}{dD_{t}},$$ (14)

where *μ*_*t*+*τ*_ is the utility in the *t*+*τ* period, *D*_*t*_ is the carbon emission in the *t* period, and *δ* is the discount factor. The Eq (14) is obtained by discounting *τ* periods.

When the temperature rise damage occurs, the temperature rise also leads to the direct utility loss, so we have the Eq (10)

$$\Delta =\sum_{\tau =0}^{\infty }\delta^{\tau }\frac{d(\Delta_{u,}{}_{t+\tau }D_{t+\tau })}{dD_{t}}-\sum_{\tau =0}^{\infty }\delta^{\tau }\frac{d[\omega_{1}\ln c_{t+\tau }]}{dD_{t}}-\sum_{\tau =0}^{\infty }\delta^{\tau }\frac{d[\omega_{2}\ln G_{t+\tau }]}{dD_{t}},$$

And when *M*_*t*_ = 1, there are Δ_*u*,*t*+*τ*_ = Δ_*u*_. According to Eq (3), we can get:

$$\Delta =\Delta_{u}-\sum_{\tau =0}^{\infty }\delta^{\tau }\frac{d[\omega_{1}\ln((1-g)y_{t+\tau })]}{dD_{t}}-\sum_{\tau =0}^{\infty }\delta^{\tau }\frac{d[\omega_{2}\ln G_{t+\tau }]}{dD_{t}},$$

According to the Eq (1), *y*_*t*+*τ*_ is expanded:

$$\Delta =\Delta_{u}-\sum_{\tau =0}^{\infty }\delta^{\tau }\frac{\omega_{1}k_{t+\tau }^{\alpha }B_{t+\tau }^{\beta }{\left[A_{t+\tau }(l_{y,t+\tau },e_{t+\tau })\right]}^{\gamma }e^{-\Delta_{y}D_{t+\tau }}(-\Delta_{y})}{(1-g)y_{t+\tau }}+\omega_{2}\sum_{\tau =0}^{\infty }\delta^{\tau }\frac{G_{t+\tau }\ln\Delta_{G}}{\upsilon G_{t+\tau }},$$

The result is shown as follows:

$$\Delta =\Delta_{u}+\frac{1}{1-\delta }\omega_{1}\frac{\Delta_{y}}{1-g}+\frac{1}{1-\delta }\frac{\omega_{2}}{\upsilon }\ln\Delta_{G}.$$ (15)

Therefore, the utility loss of carbon emissions consists of direct utility loss, output loss and green behavior loss. The third green behavior loss is included because the amount of green behavior is considered in the utility. While the temperature rise decreases people’s enthusiasm to practice green behaviors, and then abandons green behaviors because they think their behaviors have no or small significance, so that the temperature rise restrains the output of green behaviors.

In addition, when considering the marginal utility loss of current carbon emissions, we can get

$$-\frac{d\mu_{t}}{dD_{t}}=\frac{d(\Delta_{u,}{}_{t}D_{t})}{dD_{t}}-\frac{d[\omega_{1}\ln c_{t}]}{dD_{t}}-\frac{d[\omega_{2}\ln G_{t}]}{dD_{t}},$$

From Eq (3), it is concluded that:

$$-\frac{d\mu_{t}}{dD_{t}}=\Delta_{u,}{}_{t}-\frac{d[\omega_{1}\ln((1-g)y_{t})]}{dD_{t}}-\frac{d[\omega_{2}\ln G_{t}]}{dD_{t}},$$

According to Eq (1), we can obtain that:

$$-\frac{d\mu_{t}}{dD_{t}}=\Delta_{u,}{}_{t}-\frac{\omega_{1}k_{t}^{\alpha }B_{t}^{\beta }{\left[A_{t}(l_{y,t},e_{t})\right]}^{\gamma }e^{-\Delta_{y}D_{t}}(-\Delta_{y})}{(1-g)y_{t}}+\omega_{2}\frac{G_{t}\ln\Delta_{G}}{\upsilon G_{t}},$$

The result is shown as follows:

$$-\frac{d\mu_{t}}{dD_{t}}=\Delta_{u,}{}_{t}+\omega_{1}\frac{\Delta_{y}}{1-g}+\frac{\omega_{2}}{\upsilon }\ln\Delta_{G},$$

We consider the present value of utility cost *L* of current carbon emissions, which can be easily obtained from the definition:

$$L=-\sum_{\tau =1}^{\infty }\delta^{\tau }\frac{d\mu_{t+\tau }}{dE_{t}},$$ (16)

From Eq (3), it can be concluded that:

$$L=-\sum_{\tau =0}^{\infty }\delta^{\tau }\frac{d\mu_{t+\tau }}{dD_{t+\tau }}\frac{dD_{t+\tau }}{dE_{t}}=\Delta \sum_{\tau =0}^{\infty }\delta^{\tau }\frac{dD_{t+\tau }}{dE_{t}},$$

From Eq (7), it can be concluded that:

$$L=\Delta \sum_{\tau =0}^{\infty }\delta^{\tau }\pi \varepsilon \sum_{i\in L}a_{i}\frac{{\left(1-\eta_{i}\right)}^{\tau }-{\left(1-\varepsilon \right)}^{\tau }}{\varepsilon -\eta_{i}}(1-\frac{2E_{t}}{nk_{e}}),$$

The result is shown as follows:

$$L=\delta \Delta \pi \frac{\varepsilon }{1-\delta (1-\varepsilon )}\sum_{i\in L}\frac{a_{i}}{1-\delta (1-\eta_{i})}(1-\frac{2E_{t}}{nk_{e}}).$$ (17)

The utility loss per unit of emissions *E*_*t*_ during the time period *t* is decomposed into two components: the effect of temperature rises on utility and the effect of emissions on temperature rise.

For the optimal SCC, we consider the weighted utility. $\tau_{t}=\frac{\partial y_{t}}{\partial E_{t}}=L\frac{\partial c_{t}}{\partial \mu_{t}}$ can be obtained through $\frac{\partial y_{t}}{\partial E_{t}}\frac{\partial \mu_{t}}{\partial c_{t}}=L$ and $\frac{\partial \mu_{t}}{\partial c_{t}}=\frac{1}{c_{t}}=\frac{1}{(1‐g)y_{t}}$, and the optimal SCC is obtained when the climate state is *M*_*t*_ = 1.


$$\tau_{t}=L(1‐g)y_{t},$$ (18)

The above equation is expanded to $\tau_{t}(1‐g)y_{t}\delta \Delta \pi \frac{\varepsilon }{1-\delta (1-\varepsilon )}\sum_{i\in L}\frac{a_{i}}{1-\delta (1-\eta_{i})}(1-\frac{2E_{t}}{nk_{e}})$, which shows that the optimal SCC is directly proportional to the income, indicating that SCC will increase with the increase of income.

### 3.2. Carbon social cost without damage

When the climate state is *M*_*t*_ = 0, it means that the temperature rise has no impact on the economy, or the damage information of the temperature rise to the economy has not been observed. At this time, it is necessary to investigate the parameter distribution of the occurrence time of the temperature rise damage.

We set random variable *Q* to represent the utility cost of the current increase in emissions in the future. We make $L_{t}=E_{t}(Q)$ to denote the expected present value of future utility losses related to the current unit of emissions. The value of *Q* is *Q*_1_,*Q*_2_,…,*Q*_*τ*_ means SCC emitted in the period *t* in which the damage information was first observed and calculated in the period *t*+*τ*. Therefore, we assume that climate state *M*_*t*_ remains 0 in all periods before the period *t*+*τ* and then changes to 1 in period *t*+*τ*, so *Q*_*τ*_ represents the present value of marginal loss caused by carbon emissions in *t* periods and *q*_*t*_ accumulated in *τ* periods. Therefore, under the condition of *M*_*t*_ = 0, the distribution of *Q* is:

$$P(Q=Q_{\tau }|M_{t}=0)=P(M_{\tau }=1\cap M_{\tau -1}=0|M_{t}=0).$$

The premise of the above equation is that the subjective belief of the occurrence of temperature rise damage in period *t* is *μ*_*t*_, and the temperature rise damage is observed in v periods just after period *τ*. In order to find the lost corresponding cumulative distribution function *F*_*t*_(*Q*), we first discuss the probability of the occurrence of the damage in *t* periods when the initial subjective belief is *μ*_0_ (whether or not it appears for the first time in the period *t*).

First, we find out the probability of *M*_*t*_ = 1:

$$P(M_{t}=1)=P(M_{\tau }=0\cap M_{\tau -1}=0|M_{t}=0),$$

From Table 2, the probability of climate state transfer *p* is two-point distribution, and the above equation can be converted into:

$$P(M_{t}=1)=(1-\mu_{0})P(M_{t}=1|p=0)+\mu_{0}P(M_{t}=1|p=\lambda ),$$

Since *M*_*t*_ = 1 and *M*_*t*_ = 0 are mutually opposite events, we have

$$P(M_{t}=1)=1-(1-\mu_{0})P(M_{t}=0|p=0)-\mu_{0}P(M_{t}=0|p=\lambda ),$$

According to the knowledge of Bayesian statistics, we have

$$P(M_{t}=1)=1-(1-\mu_{0})\frac{P(M_{t}=0\cap p=0)}{P(p=0)}-\mu_{0}\frac{P(M_{t}=0\cap p=\lambda )}{P(p=\lambda )},$$

From Eq (11), the above equation can be simplified as:

$$P(M_{t}=1)=1-(1-\mu_{0})-\mu_{0}{\left(1-\lambda \right)}^{t},$$

The result is shown as follows:

$$P(M_{t}=1)=\mu_{0}[1-{\left(1-\lambda \right)}^{t}],$$

When the subjective belief is *μ*_*t*_, the probability of damage in the period *t*+*τ* is:

$$P(M_{t+\tau }=1|M_{t}=0)=\mu_{t}[1-{\left(1-\lambda \right)}^{\tau }],$$ (19)

The cumulative distribution function of *Q* is obtained:

$$F_{t}(Q_{\tau })=P(Q\leq Q_{\tau }|M_{t}=0)=1-\mu_{t}+\mu_{t}{\left(1-\lambda \right)}^{\tau ‐1}.$$

Considering the expected utility loss of current carbon emissions *L*_*t*_, we can know from the definition that

$$L_{t}=E_{t}\sum_{\tau =1}^{\infty }\delta^{\tau }\frac{d\mu_{t+\tau }}{dE_{t}},$$

After introducing the temperature rise damage *D*_*t*_, it can be transformed into:

$$L_{t}=E_{t}\sum_{\tau =1}^{\infty }\delta^{\tau }\frac{d\mu_{t+\tau }}{dD_{t+\tau }}\frac{dD_{t+\tau }}{dE_{t}},$$

According to Eq (7) and Eq (10), we have

$$L_{t}=E_{t}\sum_{\tau =1}^{\infty }\delta^{\tau }\Delta_{u,}{}_{t+\tau }R_{D,}{}_{\tau }M_{t+\tau },$$

By replacing *M*_*t*+*τ*_ equivalently, we can get the following result:

$$L_{t}=\Delta_{u,}{}_{t+\tau }\sum_{\tau =1}^{\infty }\delta^{\tau }R_{D,}{}_{\tau }P(M_{t+\tau }=1|M_{t}=0),$$

By using the Eq (19), it can be obtained that:

$$L_{t}=\Delta_{u,}{}_{t+\tau }\sum_{\tau =1}^{\infty }\delta^{\tau }R_{D,}{}_{\tau }\mu_{t}[1-{\left(1-\lambda \right)}^{\tau }],$$

The equivalent transformation is shown as follows:

$$L_{t}=\Delta_{u,}{}_{t+\tau }\mu_{t}\sum_{\tau =1}^{\infty }\left[\delta^{\tau }R_{D,}{}_{\tau }-\delta^{\tau }R_{D,}{}_{\tau }{\left(1-\lambda \right)}^{\tau }\right],$$

According to Eq (7), we get the following result:

$$\begin{array}{l} L_{t}=\mu_{t}[\pi \Delta_{u,}{}_{t+\tau }\delta (1-\frac{2E_{t}}{nk_{e}})\frac{\varepsilon }{1-\delta (1-\varepsilon )}\sum_{i\in L}\frac{a_{i}}{1-\delta (1-\eta_{i})}\\ \;-\pi \Delta_{u,}{}_{t+\tau }(1-\frac{2E_{t}}{nk_{e}})\delta (1-\lambda )\frac{\varepsilon }{1-\delta (1-\lambda )(1-\varepsilon )}\sum_{i\in L}\frac{a_{i}}{1-\delta (1-\lambda )(1-\eta_{i})}]. \end{array}$$

For convenience, it can be equivalent to:

$$L_{{}_{t}}=\mu_{t}L^{l}$$ (20)

It can be seen from the final result *L*_*t*_ = *μ*_*t*_*L*^*l*^ that when the climate state is *M*_*t*_ = 0, that is, when the temperature rise is in the stage of no damage to economic development, the expected utility loss *L*_*t*_ of current carbon emissions is directly proportional to the posterior probability *μ*_*t*_ of temperature rise damage. People will have an optimistic prediction of the future according to the current situation. For the undamaged stage, *μ*_*t*_ will decrease with the passage of time, so SCC described by the present value of expected utility loss $L_{t}=E_{t}(E)$ will decrease with the passage of time.

Therefore, when the climate state *M*_*t*_ = 0, the optimal SCC is shown as follows:

$$\tau_{t}=\mu_{t}L^{l}(1-g)y_{t}.$$ (21)

The expected utility loss of carbon emission is expressed as *L*_*t*_ = *μ*_*t*_*L*^*l*^, which shows that the probability of post-test of temperature rise damage *μ*_*t*_ has an indicative effect on the prediction of SCC. If climate change has no damage to economic development, the posterior probability of temperature rise damage is *μ*_*t*_→0, and the predicted value of SCC will decrease. If the posterior probability of temperature rise damage is *μ*_*t*_→1, the damage of temperature rise to economic development is on the verge. When *L*^*l*^→*L*_*t*_→*L*, the predicted value of SCC is close to Eq (18), that is, the optimal SCC when climate state *M*_*t*_ = 1.

### 3.3. Change of estimated damage in transition state

The change of SCC in the transition state is explained below. When the temperature detection value *T* exceeds the set temperature alert value $\overset{•}{T}$, it enters the transition state. From the results, the transition state can be divided into the stage of temperature rise interception and the stage of damage approaching. This section discusses the damage changes of SCC estimation under these two states.

#### 3.3.1. Estimated damage in the interception phase of temperature rise

The stage of temperature rise interception refers to that people make greater efforts to practice green behaviors to reverse the rising trend of temperature and reduce the temperature to a safe range after entering the transition state.

For The stage of temperature rise interception, people increase the intensity of green behaviors after observing the temperature alert value, so that we assume that $\mu_{t}>\frac{\mu_{t+1}}{1-\lambda }$ in this stage. Considering people’s caution, the climate change in this stage is adjusted to the following form: $Pr(p=\lambda \cap M_{t}=0)=\mu_{0}{\left(1-\lambda \right)}^{t}$. After a certain period of time, the accumulated value of temperature rise drops below the set sensitive value of temperature rise $\bar{T}$.

In the stage of damage approaching, the present value of the expected utility cost of current carbon emissions decreases gradually.


$$L_{t}>L_{t+1},\overset{•}{t}<t<\bar{t}.$$ (22)

The specific proof process is shown as follows:

$$\begin{array}{l} L_{t}=E_{t}\Delta_{u,}{}_{t}\sum_{s=1}^{i}\delta^{s}M_{t+s}\frac{dD_{t+s}}{dE_{t}}\\ \;=\Delta_{u,}{}_{t}\mathrm{Pr}(p=\lambda \cap M_{t}=0\cap \cdots \cap M_{t+i}=0)\sum_{s=1}^{i}\delta^{s}\frac{dD_{t+s}}{dE_{t}}\\ \;=\Delta_{u,}{}_{t}\mu_{t}{\left(1-\lambda \right)}^{i}\sum_{s=1}^{i}\delta^{s}\frac{dD_{t+s}}{dE_{t}}\\ \;>\Delta_{u,}{}_{t+1}\mu_{t+1}{\left(1-\lambda \right)}^{i-1}\sum_{s=1}^{i}\delta^{s+1}\frac{dD_{t+1+s}}{dE_{t+1}}\\ \;=\Delta_{u,}{}_{t+1}\mathrm{Pr}(p=\lambda \cap M_{t+1}=0\cap \cdots \cap M_{t+i}=0)\sum_{s=1}^{i}\delta^{s+1}\frac{dD_{t+1+s}}{dE_{t+1}}\\ \;=E_{t}\Delta_{u,t+1}\sum_{s=1}^{i}\delta^{s+1}M_{t+1+s}\frac{dD_{t+1+s}}{dE_{t+1}}\\ \;=L_{t+1} \end{array}$$

The conclusion is proved.

People adopt a vigilant policy and increasingly practice green behaviors after rising to the temperature rise alert value. During the period, it did not rise to the temperature sensitive value, and the temperature rise trend was suppressed, gradually returning to below the temperature safety line.

#### 3.3.2. Estimated damage in the near damage stage

When the green behaviors failed to reverse the rising trend of temperature in the interception stage of temperature rise, the temperature rise damage occurred with a certain probability after it entered the stage of damage approaching.

However, we study the stage of imminent damage from *M*_*t*_ = 0 to *M*_*t*_ = 1. We assume that the temperature $T_{\hat{t}}$ corresponding to the period $\hat{t}$ is the first period which satisfies $T_{\hat{t}}>\tilde{T}$ and $\hat{t}<\infty$.

The expected utility loss increases gradually in the near damage stage, so

$$L_{t}<L_{t+1},\hat{t}<t<\tilde{t}.$$ (23)

The specific proof process is shown as follows:

$$\begin{array}{l} L_{t}=E_{t}\Delta_{u,}{}_{t}\sum_{s=1}^{\infty }\delta^{s}M_{t+s}\frac{dD_{t+s}}{dE_{t}}\\ \;=\Delta_{u,}{}_{t}\sum_{\tau \in T}\left\{\mathrm{Pr}(M_{\tau }=1\cap M_{\tau -1}=0|M_{t}=0)\sum_{s=\tau -t}^{\infty }\delta^{s}R_{D,}{}_{s}\right\}\\ \;<\Delta_{u,}{}_{t}\sum_{\tau \in T}\left\{\mathrm{Pr}(M_{\tau }=1\cap M_{\tau -1}=0|M_{t}=0)\sum_{s=\tau -t-1}^{\infty }\delta^{s}R_{D,}{}_{s}\right\}\\ \;=\Delta_{u,}{}_{t}\sum_{\tau \in T}\left\{\mathrm{Pr}(M_{\tau }=1\cap M_{\tau -1}=0|M_{t+1}=0)\sum_{s=\tau -(t+1)}^{\infty }\delta^{s}R_{D,}{}_{s}\right\}\\ \;=\Delta_{u,}{}_{t}\sum_{\tau \in T}\left\{\mathrm{Pr}(M_{\tau }=1\cap M_{\tau -1}=0|M_{t+1}=0)\sum_{s=\tau -(t+1)}^{\infty }\delta^{s}\frac{dD_{t+1+s}}{dE_{t+1}}\right\}\\ \;=E_{t+1}\Delta_{u,}{}_{t}\sum_{s=1}^{\infty }\delta^{s}M_{t+1+s}\frac{dD_{t+1+s}}{dE_{t+1}}\\ \;=L_{t+1} \end{array}$$

The conclusion is proved.

It can be seen from Eq (23) that the temperature rise damage is determined to occur with a certain probability if the green behaviors fail to reverse the trend of temperature rise, that is, it enters the stage of damage approaching. The expected utility loss increases gradually, which means if there is no damage in this period, the temperature rise damage will occur in the subsequent period with a greater damage value.

## 4. Results and discussion

This paper considers three kinds of losses caused by temperature rise damage, but previous studies generally only considered output loss. For the convenience of comparing the results of this study with previous studies, this chapter assumes that there is only output loss. Combining with SCC model of green behaviors established in Chapter 3, this chapter will study and analyze the estimated value of SCC.

This chapter will research the European Union, in which the data of incomes comes from the research of Gerlagh R [22]. We assume that the base year is 2010. 2010 to 2200 are divided into five periods, with four probability updates in each period. Many economic and climatic parameters occur in the process of SCC analysis and calculation. Combined with the parameters in previous studies, the values of relevant parameters are shown in Table 4.

**Table 4:** Model parameters in this paper.

Parameter identification	Parameter name	Parameter value	remarks
Δ_*y*_	Output loss	4	Set according to the research [22]
*g*	Constant share of investment	0.28	According to the reference value [22] set by this paper
*δ*	Time preference rate	1%	According to the reference value [22] set by this paper
*a*	Emission share	(0.163, 0.184, 0.449)	Set according to the research [18]
*ε*	Half life of every ten years	0.183	Set according to the research [18]
*η*	Depreciation factor	(0, 0.074, 0.470)	Set according to the research [18]
*π*	Temperature sensitivity	0.0156	Set according to the research [22]
*μ*_0_	Prior initial probability	0.80	Set according to the research [22]
*α*	Initial alert times	52	For matching *μ*_0_ set by this paper
*β*	Initial stable development times	13	For matching *μ*_0_ set by this paper
*λ*	Risk transfer rate	0.77	Set according to the research [22]

Next, we will get the corresponding SCC image according to different climate states.

### 4.1. Carbon social cost of undamaged state

**Fig 3:**
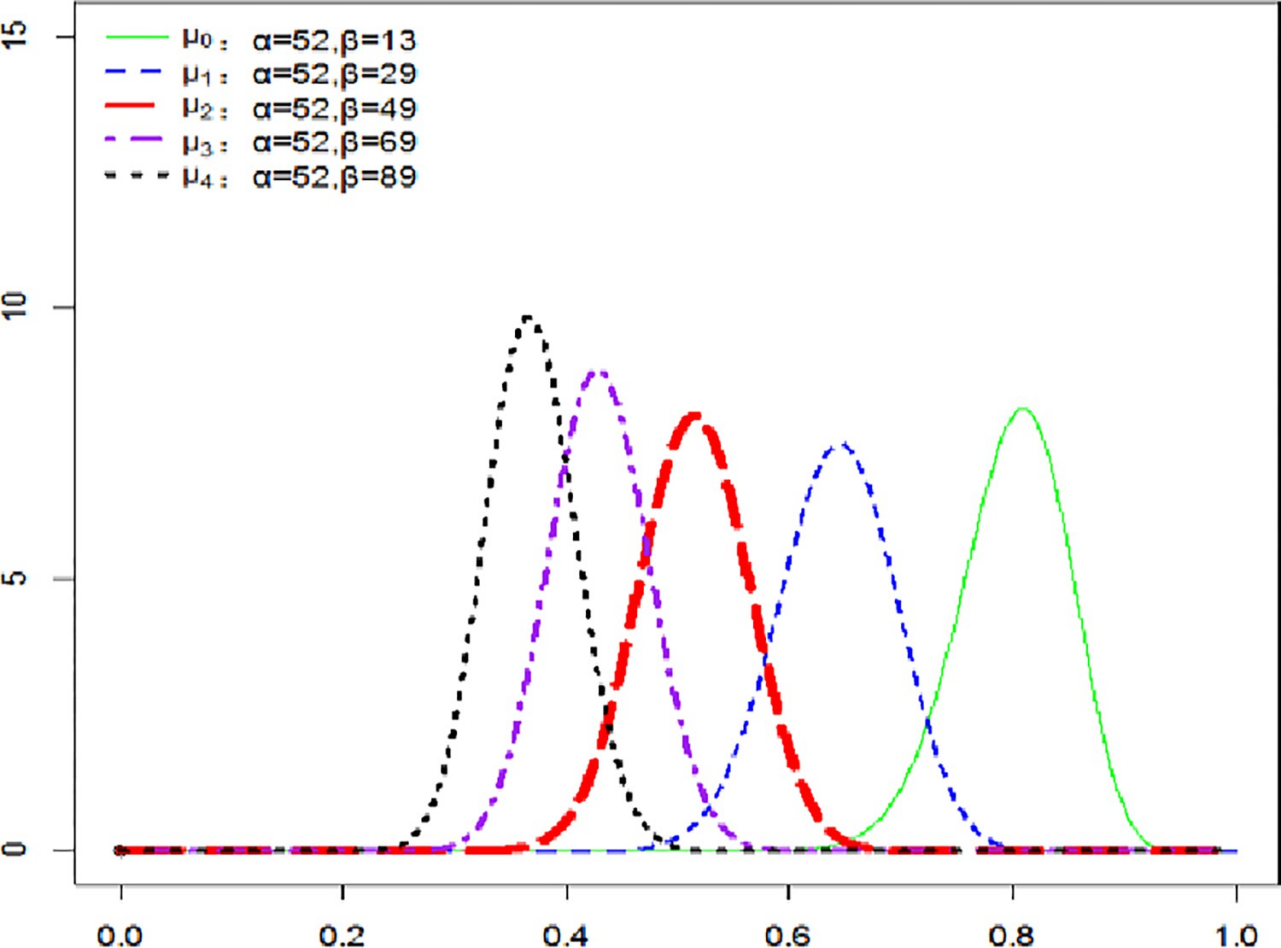
Probability update of the undamaged state.

In the undamaged state, the economic development is not damaged by the temperature rise. People’s assessment of the damage caused by the temperature rise becomes optimistic, and the probability of damage will become smaller with the passage of time. The probability of updating is shown in Fig 3.

It can be seen from Fig 3 that the prior initial probability *μ*_0_ = 0.80 has experienced 16 times of probability updating from 2010 to 2050. *α* = 52 is times of vigilance, *β* = 13+16 = 29 is times of stable development, and *μ*_1_ = 0.64 is times of probability updating. By analogy, we can get *μ*_2_ = 0.51, *μ*_3_ = 0.43, *μ*_4_ = 0.37 respectively, and the probability in the undamaged state is gradually reduced from right to left.

**Fig 4:**
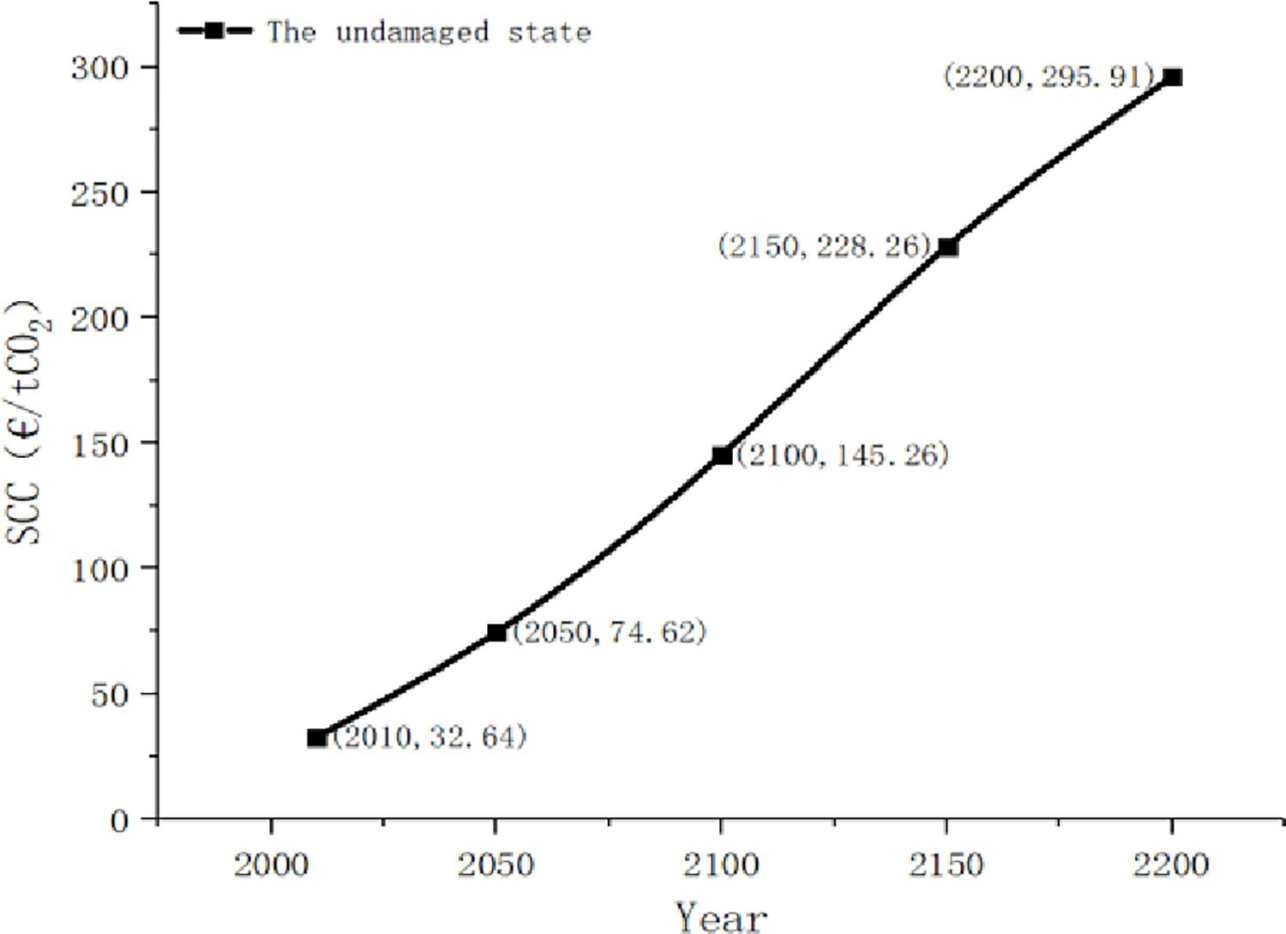
The social cost of carbon in the undamaged state.

If they were in the undamaged state in the past 200 years, people’s assessment of the economic damage caused by temperature rise became very optimistic. The probability drops from the initial 0.80 to 0.37, which is about 1/2 of the initial value. This shows that if the economy can develop steadily for a long time, the growth of SCC is extremely slow relative to the growth of income. The estimated SCC in this state is shown in Fig 4.

### 4.2. Carbon social cost of damaged state

**Fig 5:**
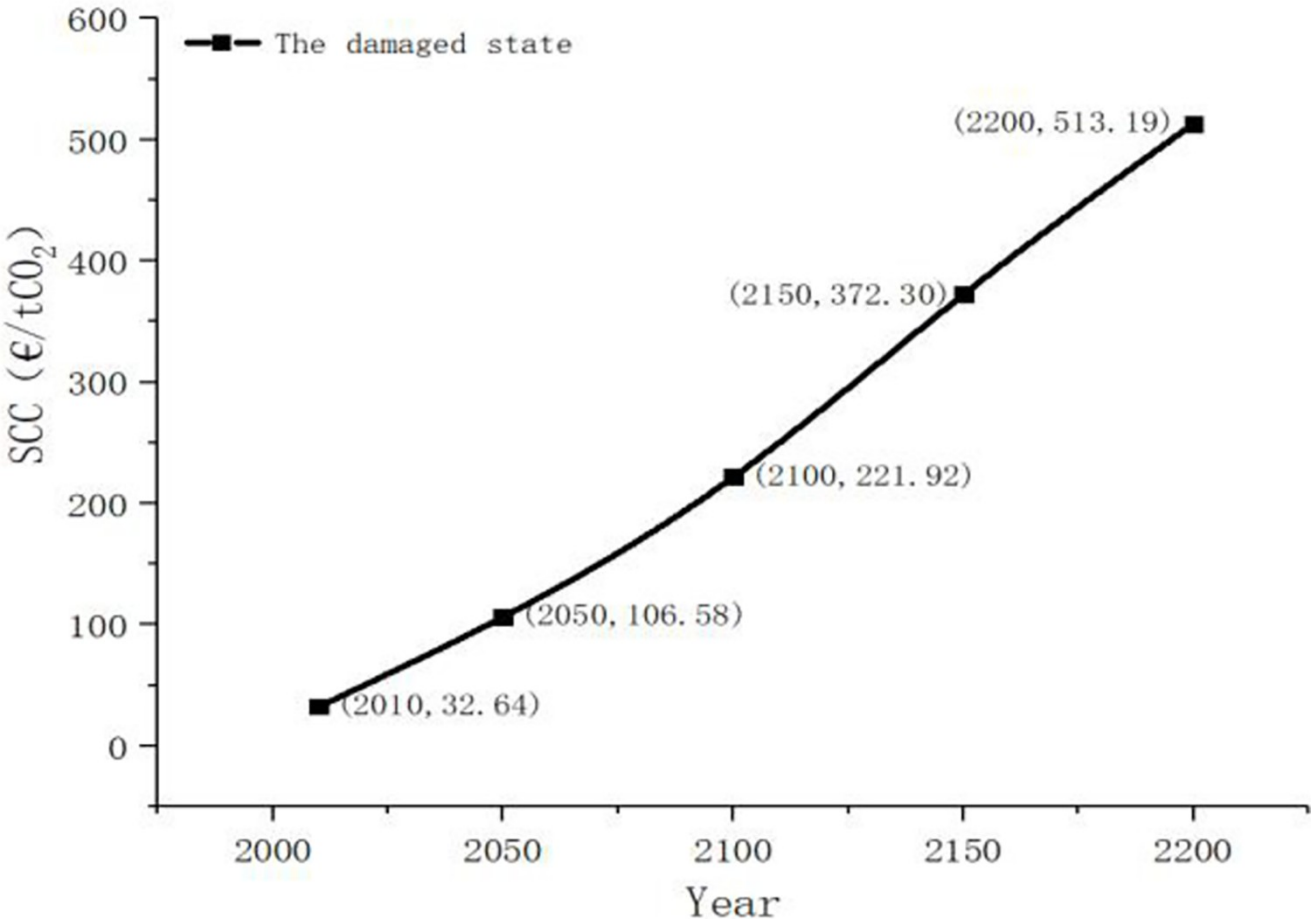
The social cost of carbon in the damaged state.

We consider SCC in the case of temperature rise damage between 2100 and 2200. We know that SCC is 32.64 euro / tCO2 and assume that it is undamaged in 2010 and the climate state will be updated to the damaged state in 2050, that is, the temperature rise will bring damage to economic development. At this time, the damage probability is updated and kept at 1, the constant coefficient is updated to 0.73, and SCC is estimated as Fig 5.

It can be seen from Fig 5 that the development of SCC between 2000 and 2050 is relatively gentle. When damage information is detected in 2050, this trend will change and the growth trend will become steeper. This also shows that when it occurs, damage will have an impact on all periods in the future.

### 4.3. The social cost of carbon in transition state

Next, we will discuss SCC in the transition period. For the convenience of comparison, we assume that 2010 is in the undamaged stage, so SCC is 32.64 €/tCO2.

#### 4.3.1. Carbon social cost at the temperature rise interception stage

**Fig 6:**
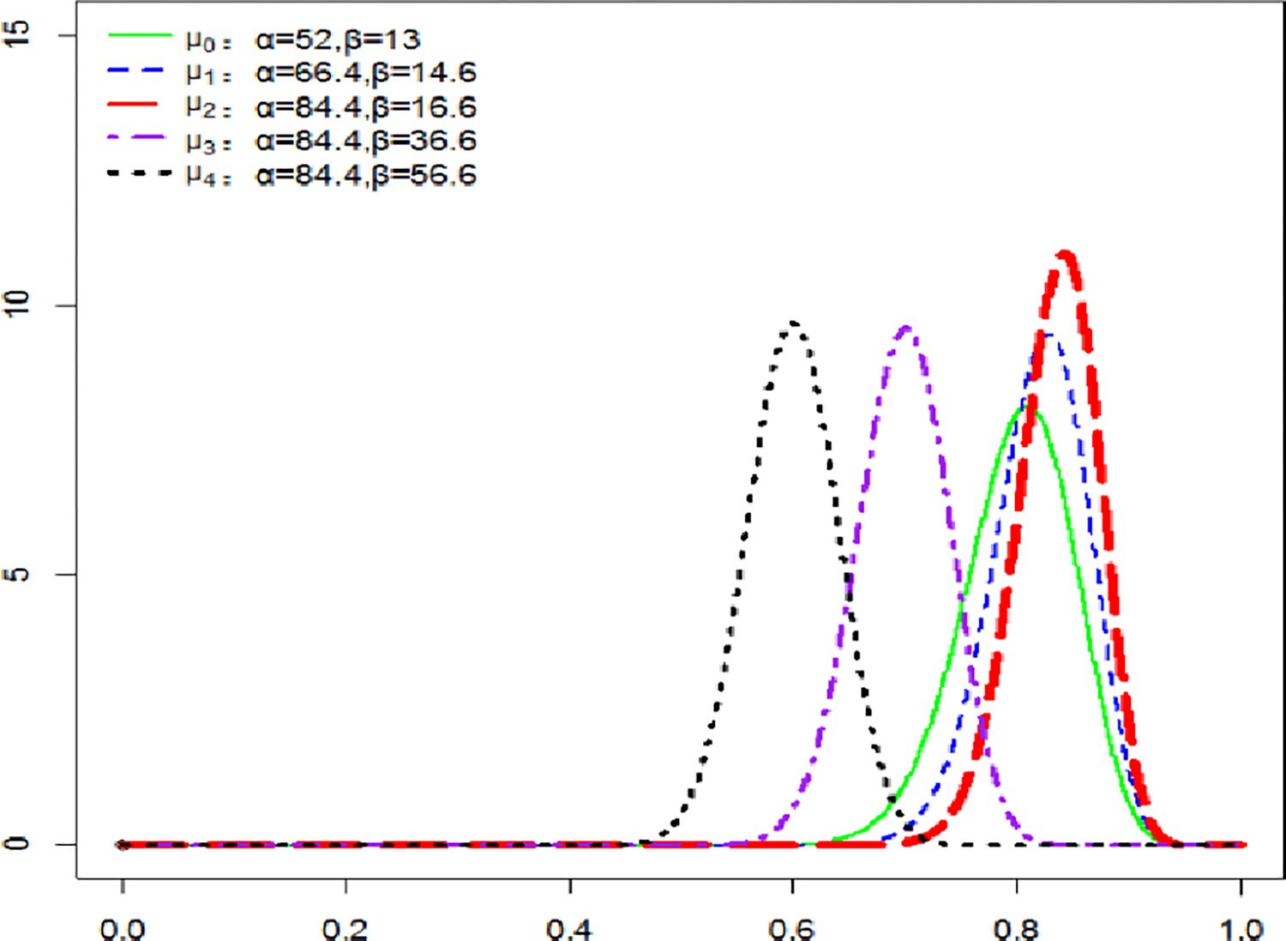
Probability update in the interception phase of temperature rise.

Firstly, we consider the temperature rise intercept phase. People adopt the alert policy when the temperature rises to the temperature rise alert value in 2010. The alert frequency *α* updates to 90% of the monitor information, and the steady development frequency *β* updates to 10% of the monitor information. In 2050, the frequency of vigilance *α* will update to 66.4, and the frequency of stable development *β* will update to 14.6. The temperature will be restored below the safety line of temperature in 2100, and the probability update will be restored in the undamaged state through increasing green behaviors and technological improvement. The distribution diagram of specific probability update is shown in Fig 6.

**Fig 7:**
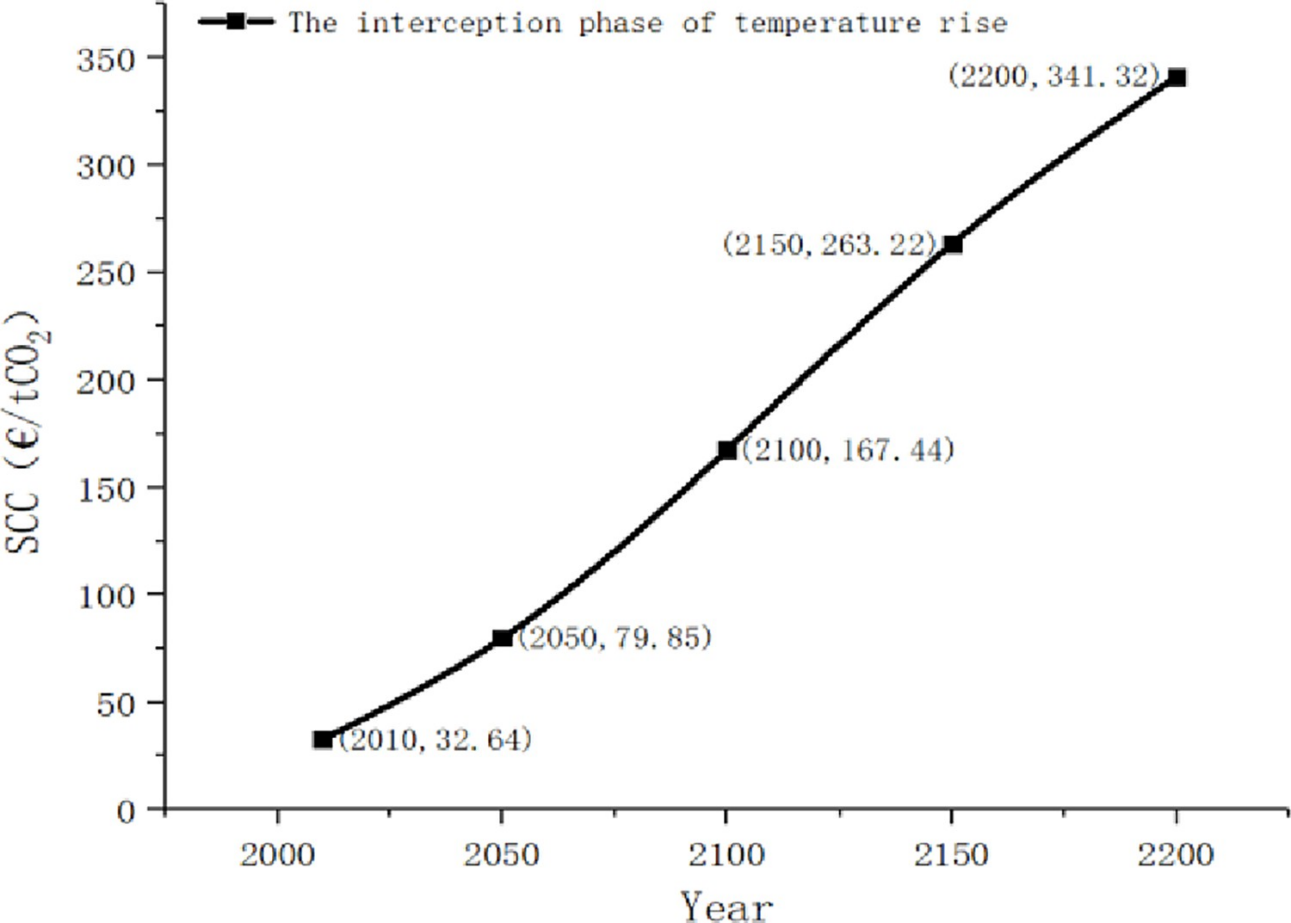
The social cost of carbon in the interception phase of temperature rise.

In Fig 6, the probability update value increases continuously under the vigilance policy from 2010 to 2100, and immediately decreases sharply after the temperature below the safe line in 2100. This also embodies people’s slack mentality after the trend of temperature rise is reversed. Then the updated value of probability will return to the undamaged state, and the probability of damage will become smaller each period. SCC obtained is shown in Fig 7.

As can be seen in Fig 7 of SCC during the temperature rise interception phase, SCC grows relatively fast during the period of vigilant policy. It compares with the slowdown of SCC in 2150, indicating that SCC will continue its growth trend after the temperature returns to below the safety line due to the increase in income, but this growth is relatively moderate and stable.

#### 4.3.2. Social cost of carbon at the stage of imminent damage

**Fig 8:**
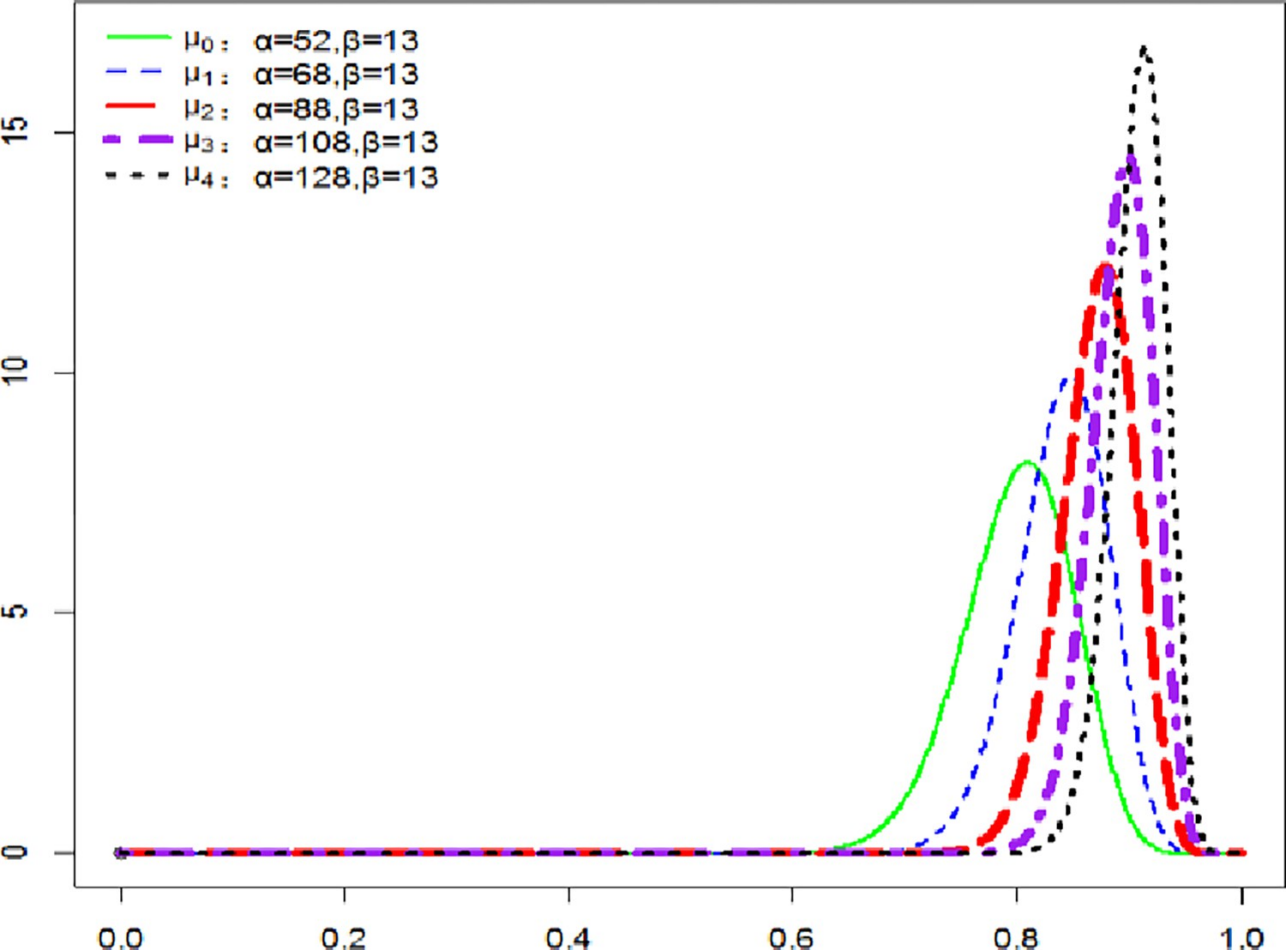
Probability update on the verge of damage state.

The imminent damage phase indicates that temperature rise damage occurs to a certain degree. In this stage, the frequency of vigilance *α* has been increasing and the frequency of stable development *β* has not changed. The specific probability which updates from 2010 to 2200 is shown in Fig 8.

In Fig 8, it is obvious that the probability update value has been increasing for this period. In this probability update mode, the probability of the recent 200 years increases by 0.11 and to 0.91. If it is always on the verge of being damaged, the damage would occur at 0.99 after 290 years.

**Fig 9:**
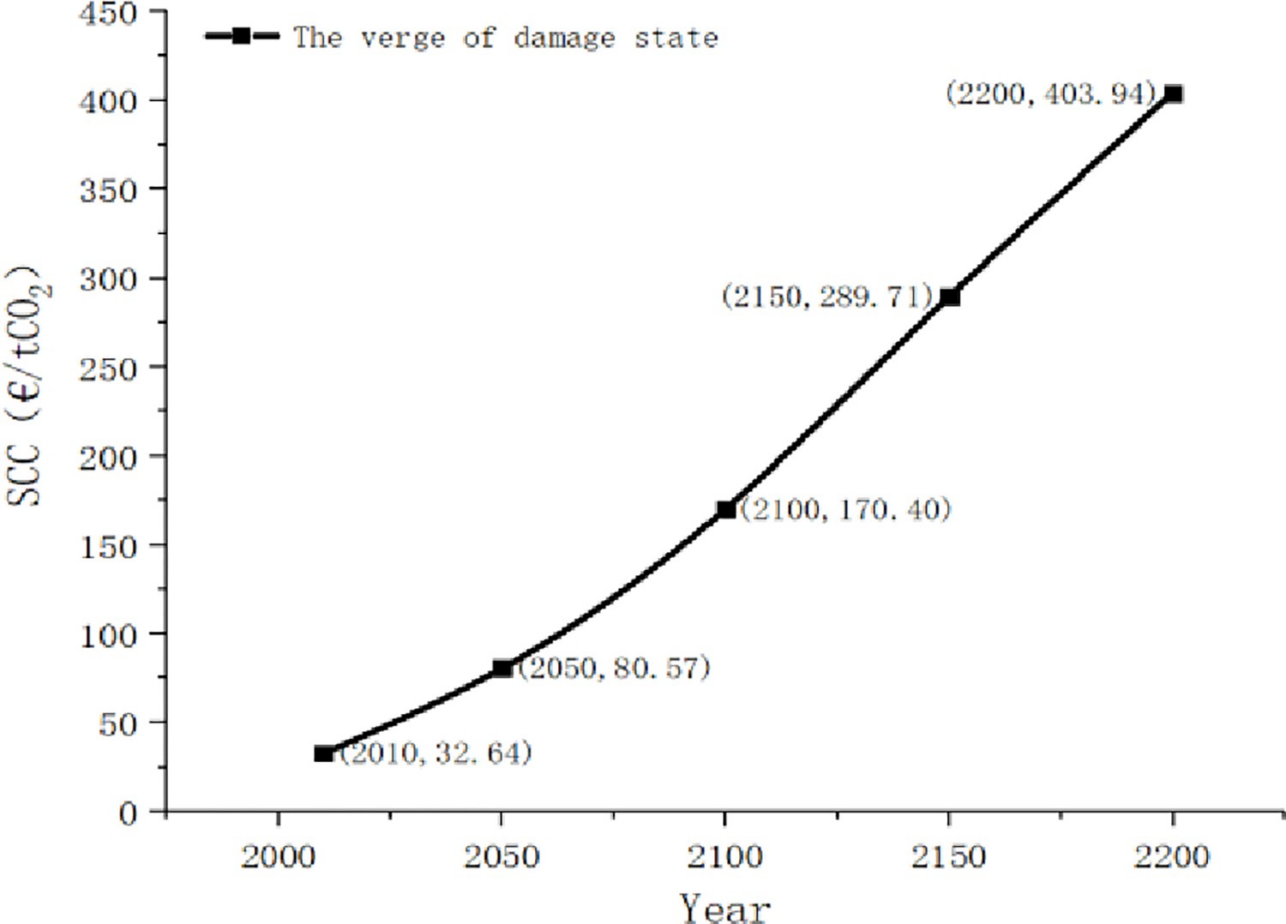
The social cost of carbon on the verge of damage state.

Similarly, the trend of carbon social cost in this stage can be calculated and plotted in Fig 9:

In the long-term stage of imminent damage, the growth of SCC is relatively fast, which is consistent with people’s crisis consciousness of imminent damage. However, the growth of SCC in this stage is relatively slower than that in the damage state.

### 4.4. Discussion

**Fig 10:**
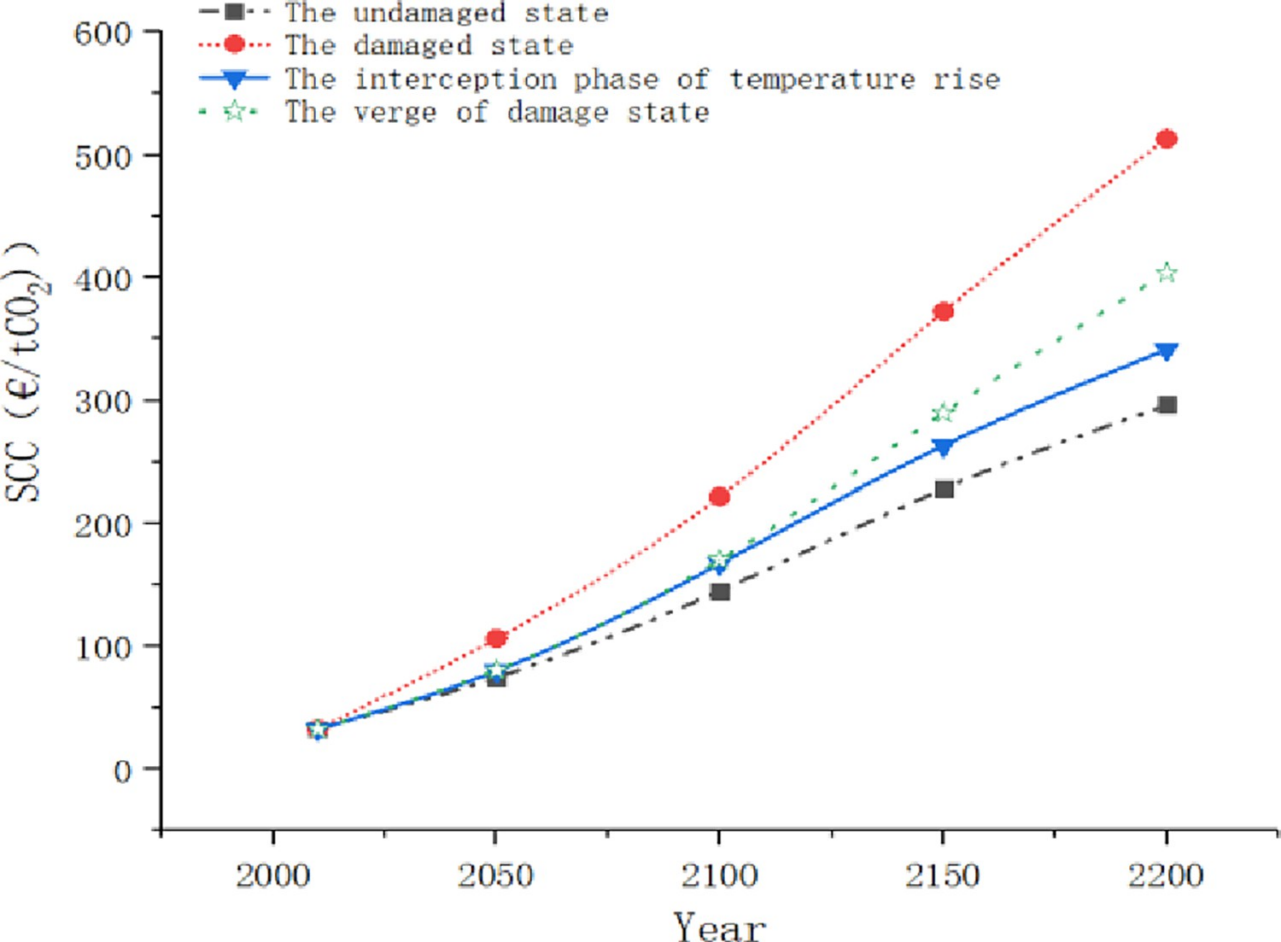
Comparison of the social cost of carbon in different climate states.

In order to more clearly analyze the impact of climate states on SCC, we compared and discussed SCC under four climate states. As Fig 10 shows, the growth trend of SCC under different climate states is greatly different when the income growth is at the same level.

SCC in the undamaged state increases most gently. In this state, the temperature rise does not cause economic losses, and green behaviors also have a positive impact on economic development, so the output value is high. On the other hand, people hold an optimistic attitude since no damage information has been observed for a long time in climate detection. So, the posterior probability of damage caused by temperature rise *μ*_*t*_ gets lower and lower to make the growth of SCC slow. The estimated value of SCC gets higher and higher mainly because of the inflation.

In the temperature rise intercept stage, people actively practice green behaviors so that carbon emissions are reduced. Therefore, the value of SCC during this period is lower than that in the stage of imminent damage, but the gap is not obvious. After the successful interception of annual temperature rise in 2100, the value of SCC will show a relatively gentle growth trend, which is more and more different from the value of SCC in the stage of imminent damage. This indicates that adopting a vigilance policy has not seen obvious benefits in a short term after the observation of climate change information, but is beneficial to the stable development of SCC in the long term.

**Fig 11:**
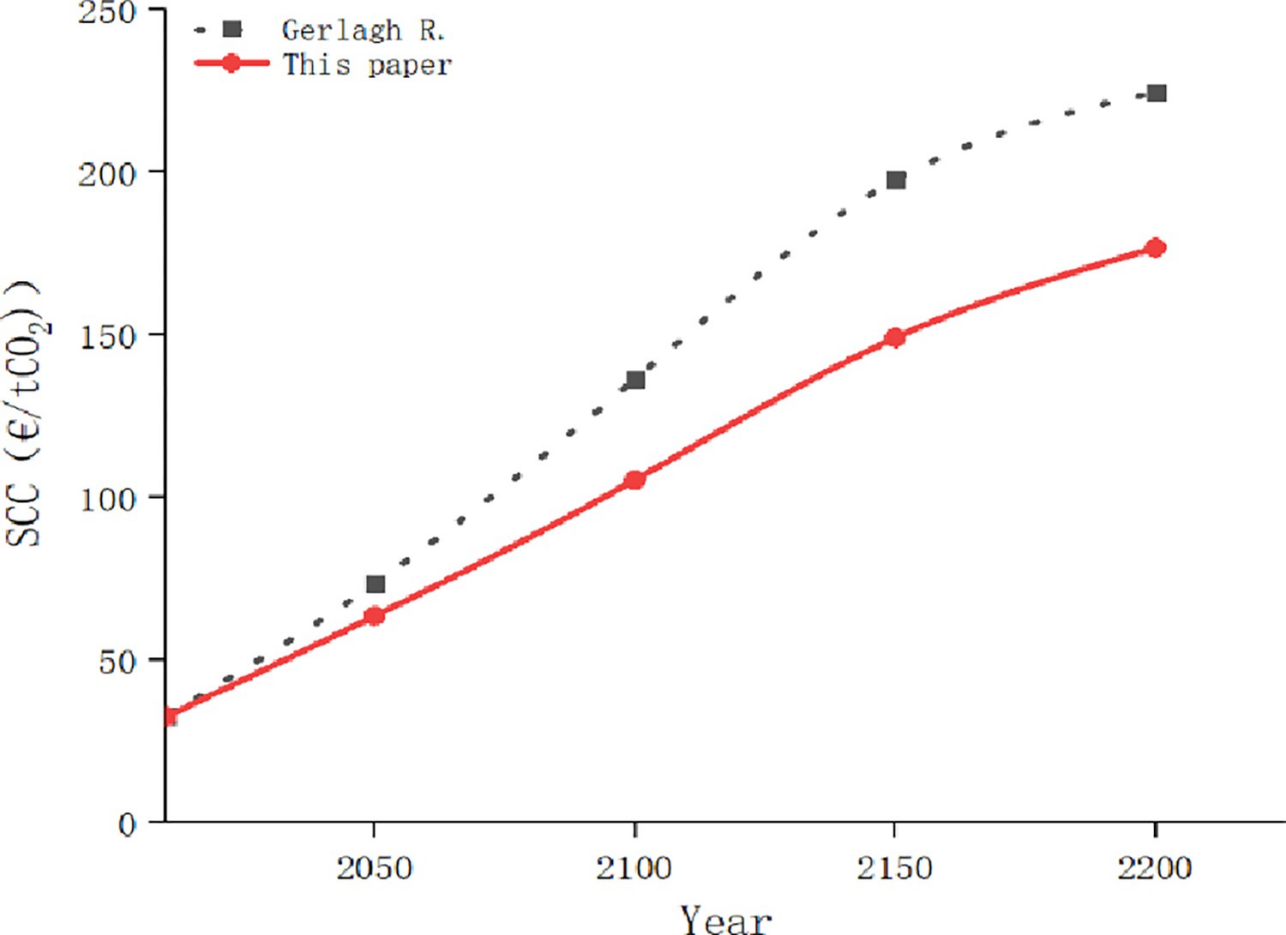
Comparison of the social cost of carbon in different climates.

We compare SCC in the undamaged state obtained in this paper with the research results of Gerlagh R and Liski [22], and the changes of the two curves are shown in Fig 11.

In Fig 11, it can be seen that the predicted value of SCC in this paper increases more gradually for the following reasons:

(1) We considered the effect of green behavior on carbon emissions and people’s optimism, both factors reduce the probability of temperature rise damage;
(2) The setting of climate state is more elaborate. The four climate states flow together smoothly. Besides, paying close attention to the state of the climate can more timely adjust policies in response to changes in the state of the climate and increase green behavior.

## 5. Conclusion

### 5.1. Main findings

Based on the perspective of people practicing green and low-carbon behaviors, the paper constructs a new model of SCC from three modules—economy, utility, and climate. Then, the paper calculates SCC under four kinds of climate states to compare SCC in different states. In addition, this article expands the utility damage caused by temperature rise into three aspects (output loss, direct utility loss, green behavior loss), and the impact of different aspects of utility damage on the social cost of carbon is discussed.

The main findings of the research are as follows:

(1) The increase of SCC in the verge of damage state is slightly larger, but it is much smaller than that in the damaged state.
(2) Green development is conducive to stabilizing the value of SCC, and close monitoring of the climate state helps to update the probability of damage in time.
(3) Output loss has a greater impact on the social cost of carbon, while direct utility loss has a weak impact. The loss of green behavior is related to the amount of green behavior.

### 5.2. Theoretical contribution

From the perspective of theoretical contribution, this study enriches the research field of SCC model. Based on the perspective of people’s green behavior, this study discusses SCC under the new situation. The values of SCC under different climatic conditions are obtained by calculation. The conclusion can provide reference for carbon price setting and can also be used for related research of social green development behavior. Therefore, this study builds a climate stochastic model and integrates Bayesian statistical methods to gain a new understanding of climate state transition. In addition, one of the main uncertainties in the estimation of the social cost of carbon is the setting of damages. This article expands the utility damage caused by temperature rise into three aspects. By giving appropriate distribution forms, the impact of different aspects of utility damage on the social cost of carbon the impact of different aspects of utility damage on the social cost of carbon has carried out new research directions and enriched relevant researches on uncertainties. It provides the basis for researchers to further study the damage function of temperature rise. The implications of this study for the practice of carbon price management are as follows: Green low-carbon behavior has a positive impact on climate status. In order to stabilize the social cost value of carbon, climate state has a great influence, so it is necessary to actively promote social green behavior and maintain a good climate state. By actively creating an atmosphere of green development consciousness, the society gives play to its subjective initiative to adjust the climate state for benign development.

### 5.3. Research limitations and perspectives

In this paper, the social cost of carbon is estimated from the perspective of people’s practice of green behavior, and the climate random probability model is used to measure whether temperature rise causes damage to the economy. Due to space limitations, the impacts of intergenerational issues, regional equity and carbon emission quota are not taken into account.

With the requirements of green development, the government, enterprises and citizens will practice green and low-carbon behaviors. Therefore, the contribution of each department to the output of green behaviors can be more accurately expressed through classified discussion. The social cost of carbon caused by climate change is of great practical significance for the research and development of new energy, economic output and future social development, and can provide policy suggestions for the formulation of emission reduction plans.

## Supporting information

S1 File Model parameters in this paper. (DOCX)
