# Supplementary material for: The social cost of carbon driven by green behaviors
Source: PLoS One. 2023 Jun 30;18(6):e0286534. doi: 10.1371/journal.pone.0286534 (PMC10313057; doi:10.1371/journal.pone.0286534)
Supplement: S1 File — (DOCX) [file pone.0286534.s001.docx]

Model parameters in this paper

| Parameter identification | Parameter name | Parameter value | remarks |
| --- | --- | --- | --- |
| $\Delta_{y}$ | Output loss | 4 | Set according to the research^[1]^ |
| $g$ | Constant share of investment | 0.28 | According to the reference value^[1]^ set by this paper |
| $\delta$ | Time preference rate | 1% | According to the reference value^[1]^ set by this paper |
| $a$ | Emission share | (0.163, 0.184, 0.449) | Set according to the research^[2]^ |
| $\varepsilon$ | Half life of every ten years | 0.183 | Set according to the research^[2]^ |
| $\eta$ | Depreciation factor | (0, 0.074, 0.470) | Set according to the research^[2]^ |
| $\pi$ | Temperature sensitivity | 0.0156 | Set according to the research^[1]^ |
| $\mu_{0}$ | Prior initial probability | 0.80 | Set according to the research^[1]^ |
| $\alpha$ | Initial alert times | 52 | For matching $\mu_{0}$ set by this paper |
| $\beta$ | Initial stable development times | 13 | For matching $\mu_{0}$ set by this paper |
| $\lambda$ | Risk transfer rate | 0.77 | Set according to the research^[1]^ |
| $L^{l}(1-g)$ | Constant coefficient | 0.68 | Set according to the research^[1]^ |

1. Gerlagh R, Liski M. Carbon prices for the next hundred years. The Economic Journal, 2018, 128(609): 728–757.
2. Gerlagh R, Liski M. Carbon Prices for the Next Thousand Years, CESifo Working Paper, 2013, 38-55.
